# The Hepatoprotective and Hepatotoxic Roles of Sex and Sex-Related Hormones

**DOI:** 10.3389/fimmu.2022.939631

**Published:** 2022-07-04

**Authors:** Linlin Xu, Yuan Yuan, Zhaodi Che, Xiaozhi Tan, Bin Wu, Cunchuan Wang, Chengfang Xu, Jia Xiao

**Affiliations:** ^1^ Department of Obstetrics and Gynecology, The Third Affiliated Hospital, Sun Yat-sen University, Guangzhou, China; ^2^ Clinical Medicine Research Institute, Department of Metabolic and Bariatric Surgery, The First Affiliated Hospital of Jinan University, Guangzhou, China; ^3^ Department of Gastroenterology, The Third Affiliated Hospital of Sun Yat-Sen University, Guangzhou, China

**Keywords:** sex hormone, chronic liver diseases, cirrhosis, mechanism, therapy

## Abstract

Most liver diseases, including acute liver injury, drug-induced liver injury, viral hepatitis, metabolic liver diseases, and end-stage liver diseases, are strongly linked with hormonal influences. Thus, delineating the clinical manifestation and underlying mechanisms of the “sexual dimorphism” is critical for providing hints for the prevention, management, and treatment of those diseases. Whether the sex hormones (androgen, estrogen, and progesterone) and sex-related hormones (gonadotrophin-releasing hormone, luteinizing hormone, follicle-stimulating hormone, and prolactin) play protective or toxic roles in the liver depends on the biological sex, disease stage, precipitating factor, and even the psychiatric status. Lifestyle factors, such as obesity, alcohol drinking, and smoking, also drastically affect the involving mechanisms of those hormones in liver diseases. Hormones deliver their hepatic regulatory signals primarily *via* classical and non-classical receptors in different liver cell types. Exogenous sex/sex-related hormone therapy may serve as a novel strategy for metabolic liver disease, cirrhosis, and liver cancer. However, the undesired hormone-induced liver injury should be carefully studied in pre-clinical models and monitored in clinical applications. This issue is particularly important for menopause females with hormone replacement therapy (HRT) and transgender populations who want to receive gender-affirming hormone therapy (GAHT). In conclusion, basic and clinical studies are warranted to depict the detailed hepatoprotective and hepatotoxic mechanisms of sex/sex-related hormones in liver disease. Prolactin holds a promising perspective in treating metabolic and advanced liver diseases.

## Introduction

Chronic liver diseases refer to a progressive deterioration of liver functions over six months. The major etiologies of chronic liver disease are genetic defect, toxin ingestion, excessive alcohol consumption, infection, autoimmune reaction, and metabolic syndromes (1, 2). Long-term inflammatory, lipid peroxidation, and necrotic insults lead to liver parenchyma destruction and scar formation (liver fibrosis). A minority of patients will progress to end-stage cirrhosis and/or hepatocellular carcinoma (3, 4). Cirrhosis, characterized by evident fibrosis and nodule formation after chronic injury, is the 11^th^ leading cause of death and the 15^th^ leading cause of morbidity around the world (5). Currently, hepatitis B virus (HBV; 31.5% in males and 24.0% in females), hepatitis C virus (HCV; 25.5% in males and 26.7% in females), and alcohol abuse (27.3% in males and 20.6% in females) are the major etiologies of cirrhosis-induced death. Obesity-related non-alcoholic fatty liver disease (NAFLD; currently 7.7% in males and 11.3% in females), which has overweight and obvious hepatic fat accumulation without a history of alcohol abuse, is also anticipated to account for increasing proportions of death in the future because of its strikingly high prevalence in the world (6). A relatively rare but important cause of cirrhosis is drug-induced liver injury (DILI), which rapidly provokes cell death, severe inflammation, oxidative stress, lipid peroxidation, and alterations in bile acid dysfunction of the liver. Some cases even have loss of immune tolerance, as well as the abnormalities of both innate and adaptive immunity (7). Even after drug cessation, a non-negligible proportion of DILI patients may progress into chronic DILI. Subsequently, more than 40% of the long-term unsolved DILI patients will develop cirrhosis (8). In the United States, the most common drugs that can induce DILI include acetaminophen, antibiotics, herbal/dietary supplements, and immunomodulatory agents (9). A recent epidemiological study indicated that in China, the leading drugs responsible for DILI are traditional Chinese medicines, herbal supplements, and antituberculosis medications (10). Since the incidence of cirrhosis and acute-on-chronic liver failure (ACLF; characterized by acute hepatic decompensation, hepatic and other key organ failure, and high short-term mortality) in DILI patients remains high, efficacious drugs that can control the progression of chronicity are urgently needed.

Sex hormones (include androgen, estrogen, and progesterone), also called sex steroids, are steroid hormones having critical functions such as reproduction, sexual development, puberty, lipid metabolism, body fat distribution, neuronal transmission, and hair growth, in the reproductive and non-reproductive systems. They are mainly produced by the gonads and adrenal glands, including the adrenal cortex, gonads (testes and ovaries), and placenta (11). The circulating and tissue levels of sex hormones are under sophisticated regulations to maintain the body homeostasis and avoid health issues such as infertility, obesity, and hair/bone loss. Several well-documented factors are able to affect the fluctuations of sex hormone levels, including aging, menstruation, stress, menopause, and medications (12). The involving roles of sex hormones in metabolic diseases received mass attention in the past decades since both basic and clinical studies found that those hormones could substantially influence the pathogenesis of or applied as novel therapies for obesity (13), type 2 diabetes (14), cardiovascular diseases (15), and NAFLD (16). Genes regulated by sex hormones are especially important for liver metabolism since the liver expresses receptors for all three sex hormones in males and females (17). In addition, there are “sexual dimorphism” for several common chronic liver diseases. For instance, females exhibit severer liver injury in alcoholic liver disease (ALD; with similar liver pathological phenotypes with NAFLD but has a history of acute or chronic alcohol abuse) and an increased risk of autoimmune liver disease than males. In comparison, hepatocellular carcinoma (HCC; the most common primary malignancy of the liver and strongly associated with cirrhosis caused by alcohol abuse and viral hepatitis) is more common in males (18). Although hormone replacement therapy (HRT) is well-tolerated by the liver, whether the therapy will change liver function and worsen the precipitated liver diseases is in debate, which is probably influenced by hormone dose, duration of therapy, alcohol drinking, smoking, genetic susceptibility, and age (19). Thus, the current review will introduce the hepatoprotective and hepatotoxic roles and mechanisms of sex/sex-related hormones and focus on the advances in elucidating the biological functions of hormone receptors. Since other sex-related hormones, including luteinizing hormone, follicle-stimulating hormone, gonadotrophin-releasing hormone, and prolactin closely coordinate with sex steroid hormones in both physiological and pathological conditions of the liver, we also summarized their involving mechanisms in chronic liver diseases.

## Sex Hormones in Liver Diseases

### Androgen

Androgens play essential roles in both sexes’ reproductive health and body metabolism. In males, testosterone is the most common androgen, which is produced by the Leydig cells of the testes, or to a lower extent, by the adrenal glands. Dihydrotestosterone (DHT), dehydroepiandrosterone (DHEA), androstenedione (A4), androstenediol (A5), and androsterone are other common types of androgens (20). In females, androgens are produced by the ovaries (testosterone), the adrenal glands (androgen precursors such as DHEA and A4), and the placenta (testosterone) during pregnancy. Androgens play an important role in female reproduction and pregnancy. Although excessive androgen clearly impairs female fertility, physiological level of androgen plays a positive role (21). Pathological conditions of females such as polycystic ovary syndrome (PCOS), obesity, and endocrinopathies (e.g. Cushing’s disease) are associated with pathologically high levels of androgens (22).

Ectopic androgen production directly induces hepatic fat accumulation, indicating a lipid regulatory role of this hormone (23–26). A low level of testosterone increases lipoprotein lipase activity, which in turn promotes triglyceride uptake into the adipocytes and subsequent visceral adiposity. Moreover, low serum DHEA levels are associated with male metabolic syndrome, possibly *via* the exacerbation of insulin resistance (27). In male rodents, androgen deficiency due to orchiectomized (ORX) also leads to hepatic steatosis (28). Mechanistically, liver lipid deposition is primarily attributed to the up-regulation of genes for *de novo* lipogenesis (DNL) (e.g. *Srebp-1c* and *Fasn*) (23). A prospective follow-up study of 942 Boston males with a median follow-up of 8.9 years reported that adiposity might influence testosterone production *via* the hypothalamus-pituitary-gonadal axis and confirmed the inverse associations between obesity and total/free testosterones (29). Therefore, in males, obesity is directly associated with low testosterone levels. Contrarily, in females, excessive androgen promotes hepatic steatosis. An important example is that androgen suppression and/or blockade significantly improves hepatic steatosis in female patients with PCOS (30). Moreover, several studies have demonstrated that excessive androgen promoted female food intake to cause obesity and metabolic dysfunction (31–33). A meta-analysis of 5,840 females (including both pre-and postmenopausal women) reported that females with higher circulating testosterone had a higher odds ratio of overweight prevalence (34). Underlying mechanisms probably include hepatic inhibition of phosphoenolpyruvate carboxykinase or up-regulation of proinflammatory mitogen-activated protein kinase 4 (MAP2K4) (35).

The contributing roles of androgens in NALFD seem to be controversial and sex-dependent (36–39). In a longitudinal analysis of 1,944 Korean men (median follow-up of 4.2 years) with repeated liver ultrasonography checks, baseline testosterone concentrations do not predict subsequent NAFLD development (40). A study investigating a cohort of 117 males shows that raising serum testosterone concentrations to normal levels by parenteral testosterone treatments reduces the serum levels of alanine aminotransferase (ALT) and aspartate aminotransferase (AST), body weight, body mass index (BMI), waist size, and improved lipid profiles (41). In comparison to males, one study of 22 postmenopausal females with biopsy-proven NAFLD indicates no significant difference in total testosterone levels with 18 matched controls (42). A large cohort study of 1,052 females in the United States identifies a novel association between free testosterone and risk of prevalent NAFLD in midlife. Importantly, this association is present even among females without androgen excess, suggesting a role of testosterone on NAFLD risk in a broader spectrum of females (43). Indeed, low androgen levels in males and high androgen levels in females facilitate NAFLD occurrence and progression. Therefore, testosterone may act as a potential new target for NAFLD treatment. In a clinical study of advanced hepatitis C-related liver disease in males, it is reported that higher serum testosterone is associated with increased risk of liver inflammation and fibrosis (44). For end-stage liver diseases, Sinclair *et al.* measure serum testosterone levels in 268 patients with cirrhosis and identify low testosterone is associated with adverse outcomes and mortality. The result significantly worsened below a total testosterone threshold of 8.3 nmol/L or a free testosterone threshold of 139 pmol/L (45). This conclusion is consistent with an observational study of 171 male cirrhotic patients in which low testosterone is found to be an independent but reliable predictor of mortality (46). Androgen signaling seems to be a potential therapeutic target of HCC since surgical castration and liver-specific androgen receptor knockout retard hepatocarcinogenesis (47). A recent study also demonstrates that pharmacologic androgen receptor antagonism with enzalutamide inhibits hepatocellular carcinogenesis in a diethylnitrosamine- (DEN-) induced HCC mouse model. More important, the upregulation of androgen receptor is only observed in portal fibroblasts and leukocytes, but not hepatocytes, implying that hepatocyte-autonomous androgen receptor signaling is not required for DEN-induced HCC (48). PD-L1 expression is negatively regulated by androgen receptor, leading to a transcriptional repression of PD-L1 and enhancement of CD8^+^T function. Thus, inhibition of androgen receptor might improve the efficacy of HCC immune-therapy to PD-L1 inhibitor (49).

Overall, decreased androgen in males or increased androgen in females may lead to metabolic disorders and end-stage liver diseases. Moderate reduction of testosterone in male is a marker of NAFLD. However, for male patients with established hypogonadism, there is no evidence that testosterone replacement therapy can induce hepatotoxicity. In females, the same focus should be on the association between ectopic androgen production and NAFLD-related risk factors/complications. Further clinical trials are needed to determine whether the reduction of physiological androgen levels will have a beneficial effect on the liver.

### Estrogen

Estrogen is one of the major female hormones, mainly secreted by the ovaries, small amounts by the liver, adrenal cortex, and breast. Estradiol is the most important form of estrogen, responsible for the regulation of female characteristics, the maturation of accessory sex organs, the menstruation-ovulation cycle, and the production of the mammary duct system (50). Serum concentrations of estrogens such as estradiol vary periodically throughout the menstrual cycle, with estradiol being the most abundant estrogen in females of childbearing age, except in the early follicular phase (51).

It is well known that the liver is a vital target tissue for estrogen signaling (52). Estrogen has a wide range of protective effects on hepatocytes. Estradiol reduces hepatic susceptibility to steatosis by strengthening cellular mitochondrial function in a substrate-specific manner (53). Moreover, 17β-estradiol (E_2_) enhances hepatocytes mitochondrial content and oxidative capacity to alleviate hepatic lipid accumulation and oxidative stress. Mechanistically, peroxisome proliferator-activated receptor gamma coactivator 1B (PGC1B), but not PGC1A, functions as a modulator of E_2_ to promote the mitochondrial biogenesis (54). Since activation of c-Jun N-terminal kinase (JNK) is a key pathological event during the development of obesity and NAFLD and E_2_ can inhibit the activation of JNK, E_2_ has been considered in NAFLD treatment in a very cautious way (55, 56).

The pathways responsible for estrogen-mediated hepatic lipid metabolism could be quite complicated and are not fully understood. Hepatocyte estrogen receptor alpha (Erα) promotes hepatic absorption of cholesterol and systemic reverse cholesterol transport, a process that is particularly important in females (57). In particular, the ability of high-density lipoprotein (HDL) to initiate reverse cholesterol transport during the female reproductive cycle is related to plasma estrogen content and hepatic ERα activity, which effectively induces cholesterol efflux from macrophages. Moreover, there is a physiologically functional cross-coupling between ERα and liver X receptor alpha (LXRα) which is another important regulator of hepatic lipid metabolism (58). Estrogen is negatively correlated with serum triglyceride (TG) level, achieved by regulating the expression of apolipoprotein A5 in the liver. Estrogen and the G protein-coupled receptor 30 coactivate protein kinase A (PKA) to enhance the expression of hepatic peroxisome proliferator-activated receptor alpha (PPARα) and hepatocyte nuclear factor 4alpha (HNF4α), thereby increasing the expression of hepatic apolipoprotein (59). Estrogen is found to directly inhibit liver inflammation since (1) postmenopause females often exhibit accumulated lipid peroxidation and inflammation in the liver (60); (2) estrogen signaling suppresses pro-inflammatory cytokine release and reactive oxygen species (ROS) production in hepatocytes (55, 61); (3) estrogen supplementation restores depressed Kupffer cell phagocytic capacity *via* the activation of Akt (62).

Estrogen is also critical in the amelioration of liver fibrosis and cirrhosis. It is reported that in a carbon tetrachloride-induced mouse liver fibrosis model, exogenous E_2_ significantly alleviated fibrosis and other liver injuries, partly *via* a restoration of miR-29a and miR-29b expression (63). Another study of male dimethyl nitrosamine (DMN) model found that estradiol treatment decreased the deposition of type I and III collagen protein, the total hepatic collagen content, and malondialdehyde (MDA), a product of lipid peroxidation (64). In high fructose diet-induced NASH-fibrosis mice models, E_2_ supplementation reversed liver cell destruction, macrophage accumulation, and hepatic stellate cell activation (65). Clinical features of a disrupted gonadal function (e.g. libido loss and reduced potency) and feminization (e.g. gynecomastia and female habitus) can be found in two-thirds of males with alcoholic cirrhosis. After transjugular intrahepatic portosystemic stent shunt (TIPS), serum E_2_ is significantly increased (with aggravated sex hormone dysbalance) in males but remain persistent in females with cirrhosis (66). In DMN-induced rat cirrhosis models, administration with E_2_ significant decreases portal pressure and increases hepatic blood flow, which are abolished by the co-treatment with an estrogen receptor antagonist (ICI-182.780) (67). In addition, estrogen stimulates the expression of nitric oxide synthase 3 (endothelial NOS, eNOS) in sinusoidal endothelial cells to provoke nitric oxide production, contributing toward a reduction in portal pressure (67, 68). Inactivated estrogen sulfates are converted to activated estrogen by the action of steroid sulfatase (STS), which is elevated in patients with chronic inflammatory liver diseases and accompanied by increased circulating estrogen levels. STS serves as a novel nuclear factor kappa-B (NF-κB) target gene to alleviate liver inflammation, partly *via* the provoked estrogen signaling (69). Estrogen is generally thought to be an anti-HCC hormone (70). Possible mechanisms include: (1) to inhibit inflammasome activation through estrogen receptor (71); (2) to repress HCC growth *via* inhibiting alternative activation of tumor-associated macrophages (72); (3) to inhibit HCC progression because of transition from pro-inflammatory to anti-inflammatory phenotype of Kupffer cell *via* the physical interaction between estrogen receptor alpha and NF-κB (73).

### Progesterone

Progesterone is a steroid hormone secreted by the granulosa luteal cells of the ovary. Ovulation, reproduction, mammary gland growth, and pregnancy maintenance are the main functions of progesterone. The primary target tissues of progesterone are the endometrium, breast, and central nervous system. In the liver, progesterone is inactivated to estradiol and excreted into the urine in combination with glucuronic acid. Progesterone can be divided into two groups according to its chemical structure: 17α-hydroxyprogesterone and 19-nortestosterone. Biological responses to progesterone are mediated by both genomic (e.g. progesterone receptor acts through specific progesterone response elements within the promoter region of target genes) and non-genomic mechanisms (e.g. non-classical progesterone receptor is activated to elicit the activation of downstream signaling) (74, 75).

Studies have shown that sex hormones have complex and variable effects on NAFLD (76). Increased progesterone level is associated with the development of systemic insulin resistance (77). This hormone is also an independent predictor of insulin resistance in adolescent girls (78). In NASH patients, progesterone use, but not estrogen use, will induce observable hepatic lobular inflammation (79). The mechanisms responsible for progesterone-induced metabolic liver injury are not characterized. A recent study indicates that deficiency of progesterone receptor membrane component 1 induces hepatic steatosis through *de novo* lipogenesis in the liver (80). Another metabolism-related study suggests that progesterone increases hepatic glucose production *via* the modulation of gluconeogenesis by progesterone receptor membrane component 1 (PGRMC1), which may exacerbate hyperglycemia in diabetes where insulin action is limited (81).

**Figure 1:**
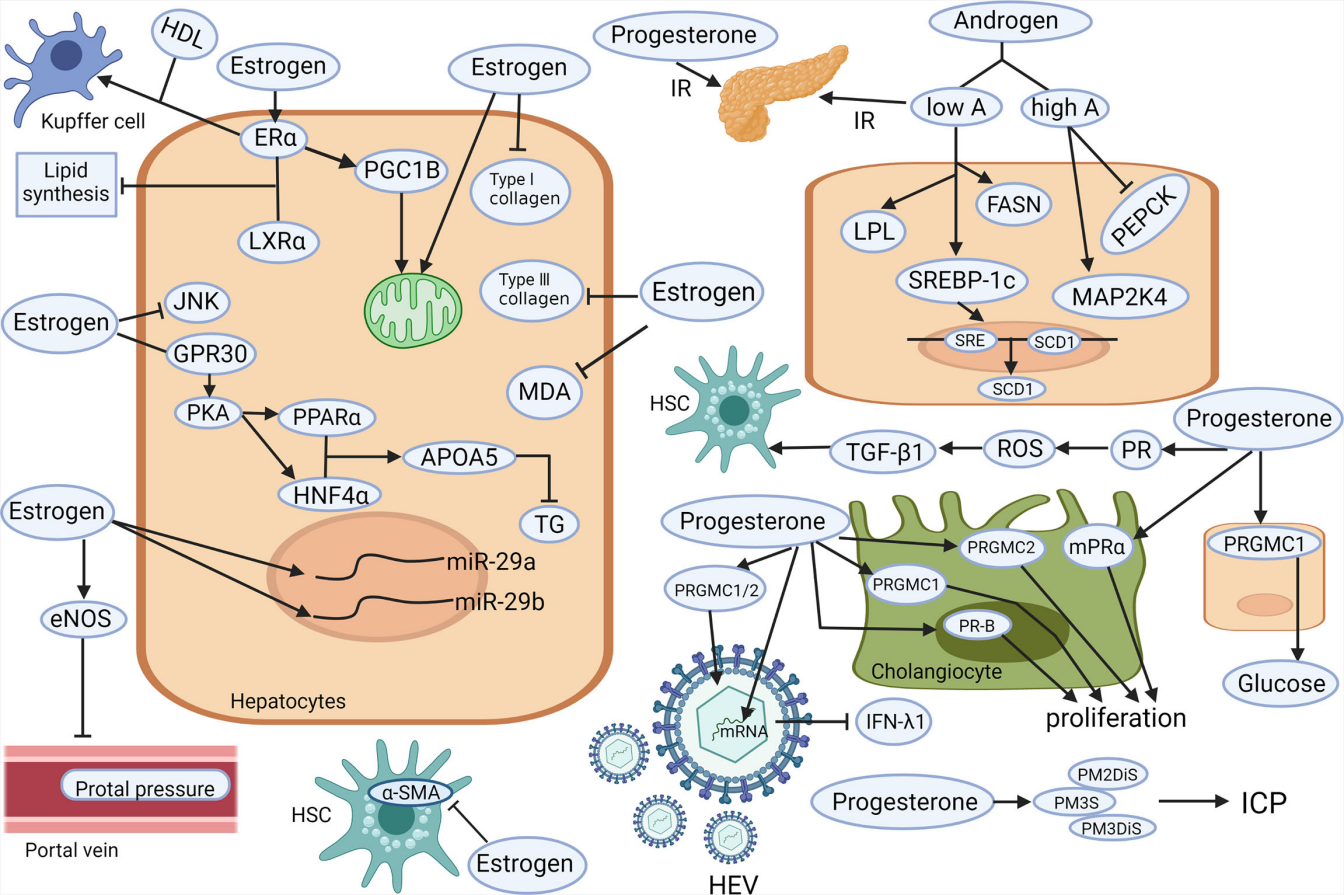
Known mechanisms of the hepatoprotective and hepatotoxic effects of sex hormones (androgen, estrogen, and progesterone) on different liver cell types. Low level of testosterone increases the activity of LPL. The up-regulation of SREBP-1c and FASN lead to liver lipid deposition and aggravated insulin resistance. Those changes, together with the down-regulation of PEPCK and up-regulation of MAPK may lead to steatosis. Estrogen increases the content and oxidation capacity of mitochondria in hepatocytes. PGC1B promotes mitochondrial biogenesis. Estrogen also inhibits the activation of JNK and GPR30 to co-activate PKA and enhance liver PPARα and HNF4α to increase APOA5 expression and reduce TG. Estrogen (via its receptor alpha) induce cholesterol efflux from Kupffer cells with HDL. Estrogen can restore the expression of miR-29a/b to reduce the deposition of type I and III collagen, MDA, and α-SMA, to reduce liver fibrosis and other types of liver damage. Estrogen can also significantly reduce portal vein pressure by stimulating eNOS expression. Elevated progesterone leads to insulin resistance, stimulates PGRMC1 to increase hepatic glucose production, stimulates PGRMC1/2 to promote HEV replication, and inhibits IFN- λ1 expression. In addition, the accumulation of progesterone metabolites (PM2DiS, PM3S, PM3DiS) will increase the risk of ICP. Progesterone stimulates PR-B, PRGMC1, and PRGMC2 to facilitate bile duct cell proliferation. It also causes ROS environment *via* its receptor signaling, resulting in TGF-β1-activated HSC. APOA5, apolipoprotein A5; α-SMA, alpha-smooth muscle actin; eNOS, endothelial nitric oxide synthase 3; ERα, estrogen receptor alpha; FASN, fatty acid synthase; HDL, high-density lipoprotein; HSC, hepatic stellate cell; GPR30, G protein-coupled receptor 30; HNF4α, hepatocyte nuclear factor 4-alpha; ICP, Intrahepatic cholestasis of pregnancy; IFN-λ1, type III interferon-λ1; JNK, Jun N-terminal kinase; LPL, lipoprotein lipase; IR, insulin resistance; LXRα, liver X receptor alpha; MAPK, mitogen-activated protein kinase; MDA, malondialdehyde; mPRα, membrane progestin receptor alpha; PEPCK, phosphoenolpyruvate carboxykinase; PGC1B, proliferator-activated receptor gamma coactivator 1B; PGRMC, progesterone receptor membrane component; PKA, protein kinase A; PM2DiS/PM3DiS/PM3S, progesterone metabolites; PPARα, peroxisome proliferator-activated receptor alpha; PR, progesterone receptor; ROS, reactive oxygen species; SCD1, stearoyl-CoA desaturase1; SRE, sterol regulatory element; SREBP-1c, sterol regulatory element-binding protein-1c; TG, triglyceride; TGF, transforming growth factor (Created with Biorender.com with a publication license).

An interesting clinical phenomenon is that females usually have worse outcomes from DILI than males. It could be partly explained by progesterone-induced immune toxic responses *via* Kupffer cells and the extracellular-signal-regulated kinase (ERK) pathway (82). Moreover, progesterone itself is reported to induce DILI in females (83). Hepatitis E is usually a self-limited liver disease with relatively good prognosis. However, during pregnancy, fulminant hepatic failure with high mortality rate is commonly observed in clinical hepatitis E virus (HEV)-infected patients. Elevated progesterone and HEV RNA levels have been observed in pregnant females with fulminant hepatic failure. Because progesterone is essential for the maintenance of pregnancy, studies on the potential role of progesterone in HEV replication and disease pathogenesis have demonstrated that in human hepatocytes, progesterone could enhance HEV replication but could not modulate HEV-induced interferon response. Loss of the progesterone noncanonical receptor, PGRMC1/2, was associated with decreased levels of HEV replication and increased levels of HEV-induced type III interferon (IFN-λ1) mRNA expression *via* the ERK pathway (84). In addition, there is a significant association between vaginal progesterone level and intrahepatic cholestasis of pregnancy (ICP). ICP is accompanied by unique maternal pruritus, abnormal liver function tests, elevated serum total bile acids, and an increased incidence of adverse fetal outcomes (e.g. intrauterine fetal death). Pregnant females who receive long-term daily vaginal progesterone treatment to prevent preterm birth are at increased risk of ICP. Progesterone metabolites (PM2DiS, PM3S, PM3DiS) are abundant during pregnancy of genetically susceptible females, leading to supersaturation of the hepatic transport system for biliary excretion of these compounds (85, 86). In animal models, progesterone induces proliferation and abnormal mitotic processes in rat liver cells. Of note, treatment with progesterone, even at pharmacologically-relevant doses, shows an increase in the percentage of binucleated hepatocytes (87). Progesterone may serve as an autocrine/paracrine mediator of cholangiocyte proliferation. Cholangiocytes express progesterone nuclear receptor (PR-B) and progesterone membrane receptors (PRGMC1, PRGMC2, and mPRα). Moreover, progesterone increases the number of bile ducts in normal rats both *in vivo* and *in vitro*, while anti-progesterone antibodies inhibit bile duct ligation-stimulated cholangiocyte growth. Thus, antiprogesterone therapy may therefore benefit patients with cholangiocyte proliferation, such as those with extrahepatic cholestasis (88). In terms of end-stage liver diseases, progesterone is reported to stimulate the production of ROS through progesterone receptor, leading to transforming growth factor (TGF)-β1 expression, hepatic stellate cells (HSCs) activation, and extracellular collagen formation (89). This phenomenon increases the possibility that progesterone can establish a favorable microenvironment for tumors and thus contribute to the development of liver cancer. PGRMC1 is considered to be a biomarker of tumor cell proliferation (90) and is strongly expressed in different kinds of cancers (91). Hepatic PGRMC1 and progesterone receptor are continuously active in the presence of high serum progesterone levels and may facilitate the chemoresistance of HCC (91, 92). Known protective and toxic mechanisms of sex hormones in the liver are summarized in **Figure 1** and **Table 1**.

**Table 1:** The involving roles and therapeutic potentials of sex hormones in liver diseases.

Liver disease	Level change of hormones	Involving mechanisms	Therapeutic potential	References
DILI	High serum estradiol reduces acute hepatotoxicity risk; Higher progesterone in pregnant females with ICP	Progesterone metabolites makes supersaturation of the hepatic transport system for biliary excretion	Anabolic androgenic steroid can induce DILI; Exogenous progesterone induces hepatic injury	(79, 85, 86, 93, 94)
Viral hepatitis	Low testosterone in HBV and HCV in males; Higher serum testosterone is associated with increased risk of HCV-related hepatitis and fibrosis; Higher progesterone in pregnant females with HEV	Androgens play immune-suppressing roles; Progesterone enhances HEV replication *via* receptor signaling	Androgen ablation therapy may be a potential therapy for HBV carriers; Estrogen can repress transcription of HBV genes	(44, 84, 95–99)
NAFLD	Low testosterone in males while higher in females; Higher progesterone in females	Androgens regulate of MAPK and hepatic metabolism; Estrogens improve liver metabolism *via* estrogen receptors; progesterone increases hepatic glucose production *via* its receptors	Testosterone and estrogen treatments improve NAFLD liver functions;	(29, 34, 35, 53–59, 81, 100, 101)
ALD	Alcohol is potentially associated with increased estrogen levels and its receptor expression	Estrogen regulates alcohol metabolism *via* Kupffer cells and inflammatory pathways	No effect for anabolic-androgenic steroids; Antiestrogen toremifene protects against ALD	(102–104)
Fibrosis and cirrhosis	Low testosterone but higher progesterone and estradiol in cirrhotic patients	Estrogen reduces collagen production and improves LSEC function; Progesterone enhances ROS and fibrogenesis	E_2_ therapy improves fibrosis and cirrhosis	(45, 46, 63–66, 89, 105)
HCC	Higher androgen and progesterone in HCC patients; Lower estrogen (in debate) in HCC patients	Estrogen protects against HCC through IL-6 restrictions; Progesterone favors carcinogenic microenvironment	Inhibition of androgen receptor represses HCC *via* inflammation and immune regulation; Estrogen therapy improves HCC	(49, 91, 92, 95, 106, 107)

ALD, alcoholic liver disease; DILI, drug-induced liver injury; E_2_, 17β-estradiol; HBV, hepatitis B virus; HCC, hepatocellular carcinoma; HCV, hepatitis C virus; HEV, hepatitis E virus; ICP, intrahepatic cholestasis of pregnancy; IL-6, interleukin-6; LSEC, liver sinusoidal endothelial cell; MAPK, mitogen-activated protein kinase; NAFLD, non-alcoholic fatty liver disease.

## Sex-Related Hormones in Liver Diseases

### Gonadotrophin-Releasing Hormone

Gonadotropin-releasing hormone (GnRH) is a decapeptide produced in the hypothalamus and secreted by scattered hypothalamic GnRH neurons in a pulsatile manner. GnRH acts on its receptor (GnRHR) on the surface of gonadotropin cells in the pituitary gland to stimulate the release of LH and FSH, which, in turn, enhance the production and release of testosterone (male testes) and estrogen (female ovaries and placenta) (108).

Experiments from murine models demonstrate that increased GnRH causes obesity after ovariectomy. As the upstream regulator of the gonad axis, GnRH stimulates fat accumulation by directly promoting the cell cycle of preadipocytes *via* the protein kinase A- cAMP-response element binding protein (PKA-CREB) pathway and increasing the FSH secretion to accelerate adipocyte differentiation in adipose tissue of female mice (109, 110). In humans, the pulsatility of serum LH levels is accepted as a GnRH pulse generator activity marker due to its short half-life (111, 112). Therefore, the exact role of GnRH in human obesity cannot be determined at present. In cirrhotic patients, disturbance in gonadotrophin secretion with inappropriately low levels of LH and FSH has been observed in amenorrheic females with alcoholic or non-alcoholic cirrhosis (113). However, these GnRH responses can only indicate the hypothalamus rather than the pituitary as the site of gonadotropin secretion disorder. Knockdown of hepatic GnRH alleviates liver fibrosis in a murine primary sclerosing cholangitis model by the downregulation of miR-200b (108). Another study confirms above findings by showing that GnRH stimulates fibrosis gene expression in HSCs in a bile duct-ligated-induced liver fibrosis rat model (114). Few studies have reported the interaction between GnRH and metabolic liver diseases, which clearly warrants further epidemiological, observational, and mechanistic investigations.

### Luteinizing Hormone

Luteinizing hormone (LH) is a glycoprotein gonadotropin secreted by adenohypophysis cells under the control of GnRH. LH can promote the conversion of cholesterol into sex hormones in gonadal cells. In females, LH stimulates the ovaries to release eggs, and its periodic surge leads to monthly ovulation. Moreover, LH stimulates the production of progesterone and estrogen, as well as the growth of the corpus luteum. In males, LH facilitates the production and release of testosterone from testicular interstitial cells in the testes.

A cross-sectional study of obese male patients in Belgium reports that NAFLD is associated with lower levels of LH, FSH, and total testosterone than controls (115). Another study with Chinese exhibits a slight but not significantly decreased LH level in NAFLD patients than that in healthy controls (116). In Chinese postmenopausal female patients, a significantly reduced LH is only observed in severe steatosis subgroup, but not in mild/moderate steatosis subgroups, when compared with that in controls (117). The direct regulatory mechanism of LH in hepatic lipid accumulation, inflammation, and cell death is largely unknown.

The LH level in seminal fluid of HCV patient is slightly, although not significantly, higher than that in healthy controls. Moreover, anti-HCV therapy does not significantly influence such level of LH in patients (118). Another similar study high larger cohort finds a slightly but not significantly lower semen LH level in HCV patients than healthy controls (119). LH and FSH levels are commonly decreased in males with advanced liver disease (120). In a Meta-analysis Of Observational Studies in Epidemiology (MOOSE) guideline report (including 21 studies with 1,274 patients), results indicate that liver transplantation (LT) improves hormonal disturbances associated with chronic liver disease by restoring circulating physiological levels of growth hormone (GH), insulin-like growth factor-1 (IGF-1), testosterone, estradiol, prolactin, FSH, and LH (121).

### Follicle-Stimulating Hormone

FSH is a glycosylated protein hormone secreted by basophils in the pituitary, because of its stimulating capacity of female follicles maturation. FSH function is mediated primarily by the FSH receptor (FSHR), which is located on the plasma membrane of hepatocytes (122). It is one of the most important hormones for development, growth, puberty, sexual maturation, and reproduction in both males and females. The level of FSH is usually low in childhood and becomes high after menopause in females. Its secretion is also in pulses with body weight change and the menstrual cycle. The determination of serum FSH is of great significance in understanding the endocrine function of the pituitary, hypothalamus, and ovary, as well as the diagnosing and treating infertility and endocrine diseases (123).

Endocrine changes during menopause, especially the dramatic increase in serum FSH levels, have a negative impact on blood lipid levels. FSH interacts with its hepatocyte receptor to decrease LDL receptor (LDLR) levels, which in turn attenuates low-density-lipoprotein cholesterol (LDL-C) endocytosis (124). After HRT, postmenopausal females with high baseline FSH levels have more significant improvement in LDL-C levels than those with low baseline FSH levels (124). Thus, HRT might be a preventive therapy in postmenopausal patients with higher basal FSH levels, and these females are encouraged to take HRT for several years after menopause. Epidemiological findings suggest that serum FSH levels are positively correlated with serum total cholesterol levels. Mechanistically, in the liver, FSH activates the Gi2α/β-arrestin-2/Akt pathway by binding to the hepatic FSHR and subsequently inhibits the binding between forkhead box protein O1 (FoxO1) and the sterol regulatory element binding protein (SREBP)-2 promoter, thereby driving 3-hydroxy-3-methylglutaryl coenzyme A reductase (HMGCR) transcription and *de novo* cholesterol biosynthesis, resulting in increased cholesterol accumulation. Therefore, blocking FSH signaling could be a novel strategy for the treatment of menopausal hypercholesterolemia, especially in perimenopausal females characterized only by elevated FSH (122).

There is an established association between FSH and NAFLD in postmenopausal females. However, it is not known whether FSH affects the risk of NAFLD in males. A community-based study of males aged 20 - 69 years observes a gradual increase in FSH with age (125). Another cross-sectional study in 444 Chinese elderly males aged 80-98 years demonstrates that high FSH levels might enhance the risk of NAFLD. Elevated FSH may be one of the possible mechanisms explaining the greater number of NAFLD subjects found in elderly males (126). Considering the age-related changes in circulating FSH levels and the prevalence of NAFLD, FSH might have a novel extragonadal role in the regulation of hepatic gluconeogenesis *via* FSHR in the liver. Moreover, there is a positive correlation between FSH and fasting blood glucose (127). FSH enhances cyclic AMP-regulated transcriptional coactivator 2-mediated gluconeogenesis *via* adenosine monophosphate-activated protein kinase (AMPK) phosphorylation regulation in the liver, leading to the pathogenesis of fasting hyperglycemia (128). In a cohort of postmenopausal females with HCV infection, there is a progressive decline in FSH from Child-Turcotte-Pugh class A to C subgroups (129). However, in male HBV-induced cirrhotic patients, such difference is not observed (130). Menotrophin is a female infertility gonadotropin treatment contains purified FSH and LH. It is reported to induce autoimmune hepatitis in a female patient after several cycles of treatment (131).

### Prolactin

Prolactin, also known as lactotropin, is a polypeptide hormone produced and secreted from the pituitary gland. The main functions of prolactin include milk production and the development of the mammary gland within breast tissues. During pregnancy, elevated prolactin promotes the growth of mammary alveoli and stimulates the breast alveolar epithelial cells to produce milk components, such as lactose, casein, and lipids (132). Notably, the level of prolactin receptor (PRLR) is suppressed on mammary glandular tissue during periods of elevated progesterone levels and is enhanced to enable lactogenesis when the serum progesterone level drops (133).

**Figure 2:**
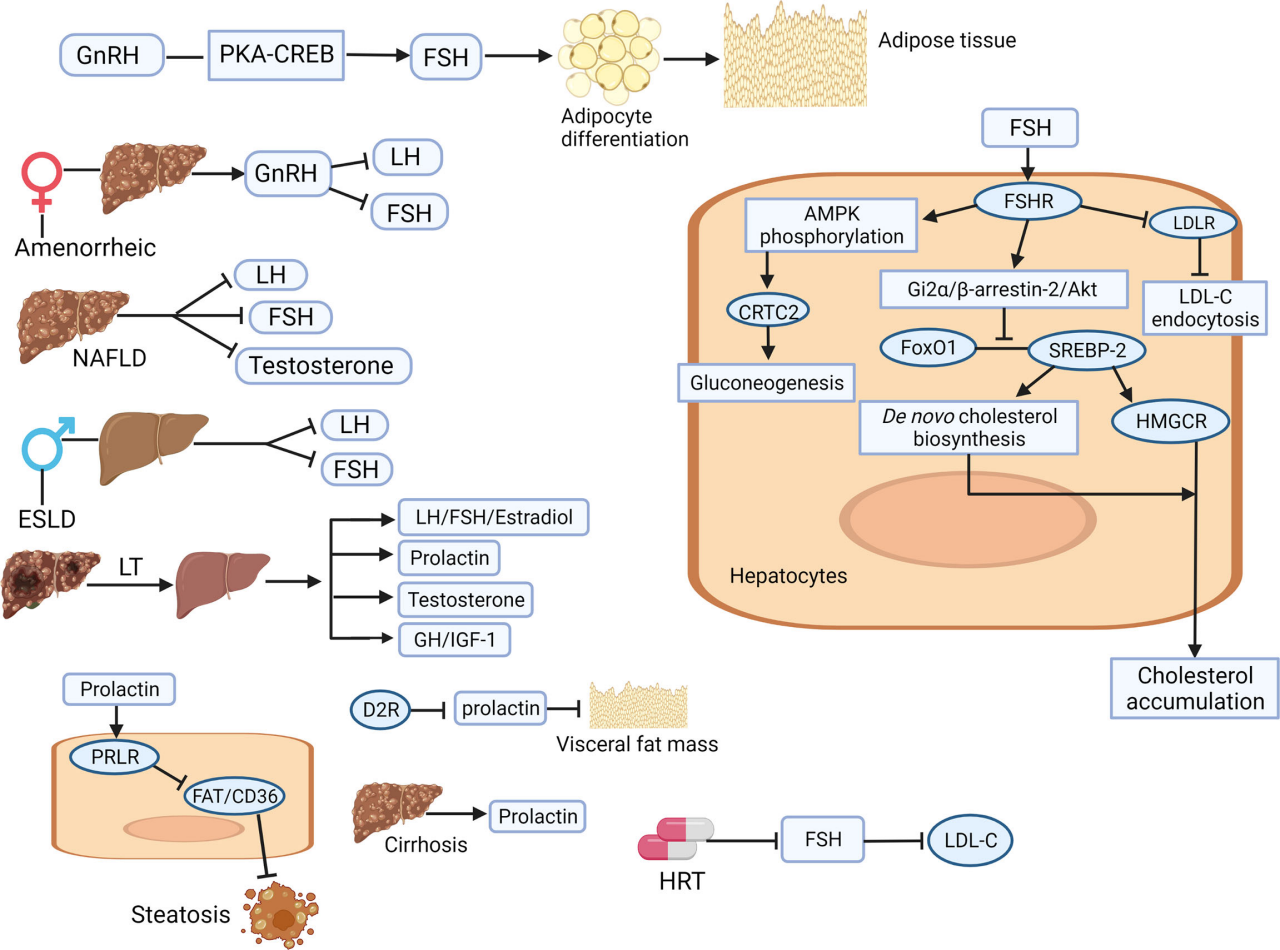
Illustration of the involving mechanisms of sex-related hormones (gonadotrophin-releasing hormone, luteinizing hormone, follicle-stimulating hormone, and prolactin) in liver physiology and pathology. GnRH increases the secretion of FSH through the PKA/CREB pathway, accelerates the differentiation of adipocytes in adipose tissue, which finally lead to hepatic fat accumulation. In amenorrhoea females with cirrhosis, abnormal GnRH secretion leads to low LH and FSH levels. FSH interacts with FSHR to reduce the level of LDLR, weaken the endocytosis of LDL-C, and lead to the increase of circulating LDL-C. After HRT treatment, FSH is inhibited and LDL-C content is improved. FSH activates Gi2 by binding to liver FSHRα/β-Arrestin-2/Akt pathway, which subsequently inhibits the binding between FoxO1 and SREBP-2, drives HMGCR transcription and *de novo* cholesterol biosynthesis, resulting in increased cholesterol accumulation. Liver transplantation can improve the hormone disorder related to chronic liver disease by restoring the circulating physiological levels of estradiol, FSH, LH, prolactin, testosterone, GH and IGF-1. Inhibition of prolactin by D2R activation leads to reduction of visceral adipose tissue. Prolactin improves hepatic steatosis through PRLR down regulation of FAT/CD36. Prolactin levels are significantly increased in patients with liver cirrhosis. Akt, protein kinase B; AMPK, adenosine monophosphate-activated protein kinase; CREB, cAMP response element binding protein; ESLD, End-stage liver disease; FAT, fatty acid translocase; FoxO1, forkhead box protein O1; FSH, follicle-stimulating hormone; FSHR, FSH receptor; GH, growth hormone; GnRH, gonadotrophin-releasing hormone; HMGCR, 3-hydroxy-3-methylglutaryl coenzyme A reductase; HRT, hormone-replacement therapy; IGF, insulin growth factor; LDL, low-density lipoprotein; LDL-C, low-density-lipoprotein cholesterol; LDLR, LDL receptor; LH, luteinizing hormone; LT, liver transplantation; PKA, protein kinase A; PRLR, prolactin receptor (Created with Biorender.com with a publication license).

Prolactin has a major role in determining the deposition and mobilization of fat. Thus, it is suggested that in both adults and children, increased body weight alters the secretion of prolactin, possibly due to hyperinsulinemia-induced hypothalamic-pituitary dysfunction. In obese females, enhanced prolactin release is in proportion to the size of the visceral fat mass. A possible explanation is reduced dopamine D2 receptor (D2R) availability in the brain, since prolactin is inhibited by D2R activation (134). After the loss of 50% of overweight, such elevated prolactin secretion rate in obese females is significantly blunted, along with increased dopaminergic signaling (135). However, a large cross-sectional study assessing serum prolactin levels in 344 males and females obese subjects’ samples one year after gastric bypass surgery finds no significant association between basal prolactin levels and the degree of obesity or between the change of systematic prolactin level and weight loss. Thus, there does not seem to be a significant role of prolactin in the pathophysiology of obesity (136). Since obesity is associated with higher NAFLD incidence, whether prolactin has hepatoprotective roles in NAFLD received mass attention. A recent clinical study with 859 adults (456 patients with NAFLD and 403 controls without NAFLD) identifies that circulating prolactin levels and hepatic *Prlr* gene expression levels are lower in NAFLD patients than those of healthy controls (in both sexes). Moreover, in cell models, prolactin ameliorates hepatic steatosis *via* PRLR and fatty acid translocase (FAT)/CD36, an important hepatic transporter of free fatty acid (137). Thus, prolactin level, body mass index, alanine aminotransferase, HDL cholesterol, and HbA1cA are included in a new noninvasive model for the prediction of NAFLD presence (138). In terms of ALD, since acute and repeated alcohol ingestions sharply rise plasma prolactin levels and decrease plasma testosterone levels in male volunteers (139) and ethanol induces hyperprolactinemia lactotrope growth in female rats (140), it is speculated that increased prolactin is an endogenous protective mechanism to alleviated injury of the liver, and possibly other ethanol-targeted tissues, *via* unknown pathways. Another study finds a drastic increase in serum prolactin in cirrhotic patients than in healthy control, regardless of hepatic encephalopathy presence, and a cut-off value (50 ng/ml) is capable to predict the mortality (141). This result is consistent with a clinical report including 114 male cirrhotic patients whose increased prolactin level is parallel to growing cirrhosis severity (142). In HCC, prolactin prevents cancer growth by restricting innate immune activation of c-Myc in mice (143). A recent study reports that prolactin upregulated female-predominant cytochrome P450 genes in female mice and downregulated male-predominant 450 genes in male mice, which may explain the abnormal drug metabolism and DILI during pregnancy and lactation (144). In conclusion, increased secretion of prolactin from the pituitary seems to be beneficial for the development of both metabolic and end-stage liver diseases. Compared to other sex/sex-related hormones, prolactin holds a promising perspective in exogenous hormone therapy for those diseases. However, molecular mechanisms and well-designed RCTs are still warranted to be investigated. Known protective and toxic mechanisms of sex-related hormones in the liver are summarized in **Figure 2** and **Table 2**.

**Table 2:** The involving roles and therapeutic potentials of sex-related hormones in liver diseases.

Liver disease	Level change of hormones	Involving mechanisms of hormones	Therapeutic potential of hormones	References
DILI	Acetaminophen use is inversely associated with prolactin but no association with LH/FSH	Prolactin promotes liver regeneration *via* IL-6/SOCS3 pathway	GnRH agonist causes hepatotoxicity; Menotrophin induces DILI;	(131, 145–148)
Viral hepatitis	High LH in male HBV patients	Unknown	Unknown	(149)
NAFLD	Lower GnRH, FSH, and prolactin in both sexes’ patients;	GnRH stimulates fat accumulation through PKA-CREB; Increased FSH secretion accelerates adipocyte differentiation; FSH modulates hepatic gluconeogenesis *via* FSHR and AMPK; Prolactin protects steatosis *via* PRLR and FAT and CD36	Prolactin therapy may improve NAFLD	(109, 115, 116, 127, 128, 137, 138)
ALD	Higher LH and prolactin but lower testosterone in box sexes	Prolactin protects ALD *via* unknown pathway	Prolactin therapy may improve ALD	(139, 140, 150)
Fibrosis and cirrhosis	Lower LH and FSH but higher prolactin in cirrhotic patients of both sexes	Knockdown of GnRH improves fibrosis *via* miR-200b inhibition	Prolactin therapy improves fibrosis by inhibiting GnRH	(108, 113, 114, 120, 141, 142, 151)
HCC	Increased LH and FSH but decreased prolactin in HCC patients of both sexes	Prolactin prevents HCC by restricting innate immune activation of c-Myc	GnRH immunogen vaccination inhibits liver tumor; Prolactin therapy may retard HCC	(143, 152, 153)

ALD, alcoholic liver disease; AMPK, adenosine monophosphate-activated protein kinase; CD36, cluster of differentiation 36; CREB, cAMP response element binding protein; DILI, drug-induced liver injury; E_2_, 17β-estradiol; FSH, follicle-stimulating hormone; FSHR, FSH receptor; GnRH, gonadotrophin-releasing hormone; HBV, hepatitis B virus; HCC, hepatocellular carcinoma; IL-6, interleukin-6; LH, luteinizing hormone; MAPK, mitogen-activated protein kinase; NAFLD, non-alcoholic fatty liver disease; PKA, protein kinase A; PRLR, prolactin receptor; SOCS-3, suppressor of cytokine signaling 3.

## Gut-Liver Axis Regulation by Sex/Sex-Related Hormones

**Figure 3:**
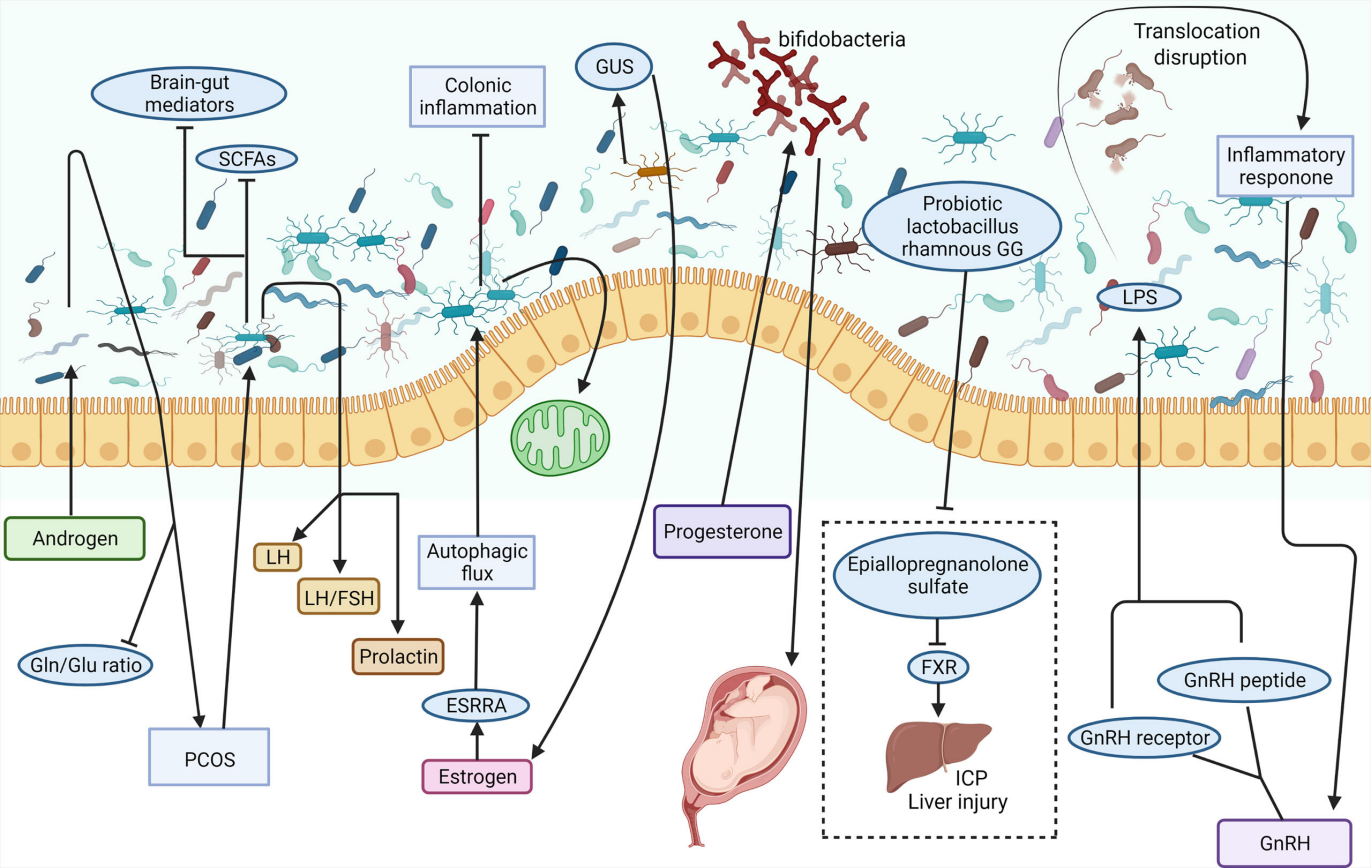
Possible involving mechanisms of sex/sex-related hormones in the regulation of the gut-liver axis. The intestinal microbiome is a complex microbial ecosystem. Androgen induced dysbacteriosis may aggravate PCOS and reduce the circulating Gln/Glu ratio. The study on the effects of intestinal microorganisms on gut brain mediators and sex hormones in patients with PCOS showed that prolactin, LH and LH/FSH ratio increased significantly, while brain-gut mediators and SCFAs decreased. Estrogen can improve colitis and protect mitochondrial function by ESRRA-mediated autophagy. One of the main regulators of circulating estrogen is the intestinal microbiome *via* the secretion of GUS. Progesterone promotes the growth of bifidobacteria in the third trimester of pregnancy and transmits it to newborns. Probiotic *Lactobacillus rhamnosus* GG can prevent epicallopregnanolone sulfate-mediated FXR activation and bile acid synthesis, so as to reduce liver bile acid accumulation and liver injury in ICP patients. Dysfunction of intestinal microbiota may lead to LPS leakage, inflammatory response, and GnRH secretion abnormality. ESRRA, estrogen related receptor alpha; FSH, follicle-stimulating hormone; FXR, farnesoid X receptor; GnRH, gonadotrophin-releasing hormone; GUS, β-glucuronidase; ICP, intrahepatic cholestasis of pregnancy; LH, luteinizing hormone; LPS, lipopolysaccharide; PCOS, polycystic ovary syndrome; SCFA, short chain fatty acid (Created with Biorender.com with a publication license).

The gut microbiome is a microbial ecosystem involved in nutrient acquisition and energy metabolism of the host (154). Sex plays an important role in the composition diversity of the gut microbiota (155, 156). The alpha diversity of the gut microbiota is higher in females than in males (157, 158), with differences occurring at the onset of puberty, suggesting that sex hormones cause important composition changes of the gut microbiome (159, 160). Hyperandrogenism is a key factor in impaired follicular development and metabolic disorders in PCOS. In the animal model, intestinal dysbacteriosis is reproduced in DHEA-induced PCOS-like rats. Antibiotic mixtures can be used to eliminate the gut microbiota during DHEA treatment. However, depletion of the gut microbiota does not prevent the development of the PCOS phenotype in DHEA-treated rats. The DHEA type intestinal microflora transplanted into a pseudo-sterile recipient cause disorders of hepatic glycolipid metabolism and reproductive hormone imbalance. These findings suggest that androgen-induced dysbacteriosis may exacerbate metabolic and endocrine dysfunction in PCOS (161). Males are generally more vulnerable to glucose imbalance and diabetes than females. It is revealed that the depletion of the mice gut microbiome largely eliminates sexual dimorphism in glucose metabolism. Glucose tolerance in male mice is more evidently influenced by the gut microbiome than in female mice. Androgen treatment improves glucose tolerance and insulin sensitivity, in part by modulating the gut microbiome, leading to sexual dimorphism in glucose metabolism. Androgens also regulate circulating glutamine and glutamine/glutamate (Gln/Glu) ratios partly *via* the actions of gut microbiome. Exogenous glutamine supplementation may increase insulin sensitivity *in vitro* (162). In terms of estrogen, its related receptor alpha (ESRRA) acts as a key regulator of gut homeostasis by activating autophagic flux and controlling host gut microbiota to improve colonic inflammation. In animal models, ESRRA-deficient mice exhibit a distinct gut microbiota composition and significantly higher microbial diversity compared to wild-type mice. ESRRA promotes gut homeostasis through autophagy activation and gut microbiota control to protect the host from harmful inflammation and mitochondrial dysfunction (163). One of the major regulators of circulating estrogens is the gut microbiome, which modulates estrogen by secreting β-glucuronidase (GUS), an enzyme that breaks down estrogen into its active form. When this process is impaired by dysbacteriosis in the gut, the reduction in deconjugation results in a decreased level of circulating estrogens (164). Changes in circulating estrogens may lead to the development of several diseases (obesity, metabolic syndrome, cancer, endometrial hyperplasia, endometriosis, PCOS, infertility, cardiovascular disease, and cognitive function). Modulation of microbiome composition has been shown to alleviate many estrogen-regulated disease progressions (164). Pregnancy is accompanied by changes in the microbiome, and progesterone, the main pregnancy hormone, is found to directly regulate the intestinal microbial composition during pregnancy, such as promoting the growth of bifidobacteria species (probiotics that live in the intestines) in late pregnancy, in order to transmit them to newborns (165). In the serum of ICP patients, the level of a progesterone metabolite, epiallopregnanolone sulfate is significantly elevated, which can inhibit farnesoid X receptor (FXR)-mediated bile acid export and synthesis. Administration with probiotic Lactobacillus rhamnosus GG prevents epiallopregnanolone sulfate-induced hepatic bile acid accumulation and liver injury, possibly mediated by hepatic FXR activation (166). GnRH is associated with gut motility through GnRH receptors signaling, primarily in cells of parasympathetic ganglion and myenteric plexus of the enteric nervous (167). There is a bidirectional relationship between intestinal flora and GnRH/GnRH receptor signaling axis (168). The potential interaction between GnRH and the gut microbiota has been suggested through a lipopolysaccharide (LPS)-induced proinflammatory pathway (169). Disruption of gut microbiota or large bacterial translocations may lead to greater circulation alterations in LPS, inflammatory responses, and GnRH production (170). A study monitoring the effects of the probiotic *Bifidobacterium lactis* V9 on the gut microbiome, gut-brain mediators, and sex hormones in 14 PCOS patients shows significant higher levels of prolactin, LH and LH/FSH ratio when compared with 9 volunteers. The levels of sex hormones, brain-gut mediators (e.g. ghrelin) and intestinal short-chain fatty acids (SCFAs) are conversely regulated (171). In a rat model, dietary flaxseed oil (FO) intake improves the disturbance of estrous cycle and ovarian morphology, as well as the disorder of sex/sex-related hormones, including testosterone, estrogen, progesterone, and LH/FSH, body weight, dyslipidemia, and insulin resistance. One of the major mechanisms is through the sex steroid hormone-microbiota-inflammatory axis (172). Possible involving mechanisms of sex/sex-related hormones in gut-liver axis regulation are illustrated in **Figure 3**.

## Hepatic Safety Issue of Gender-Affirming Hormone Therapy in Transgender Populations

**Figure 4:**
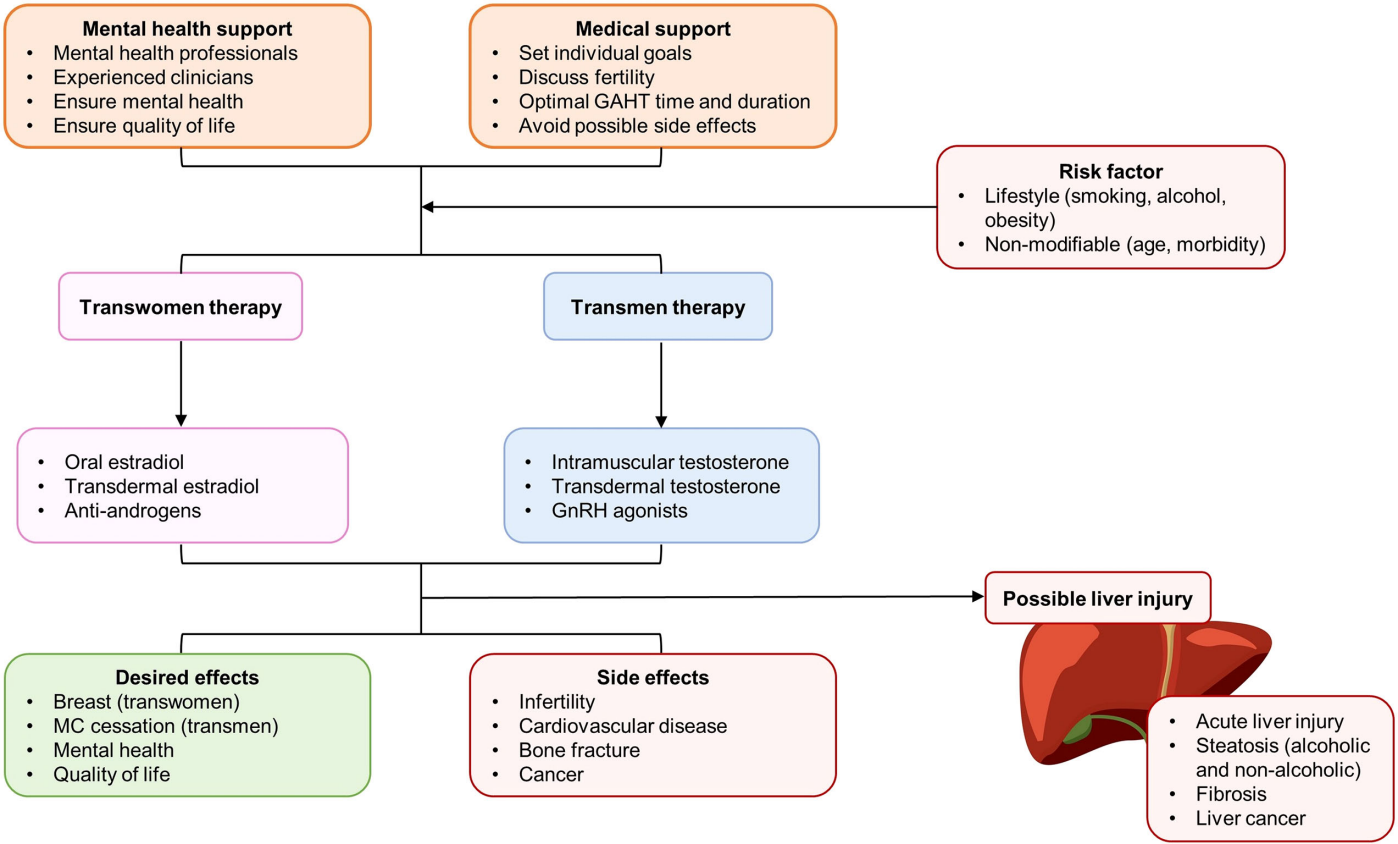
Patient care, therapy selection, and balance between desired effects and potential side effects (with special emphasis in the liver) of gender-affirming hormone therapy (GAHT) for transgender populations. AMPK, D2R, dopamine D2 receptor; GnRH, gonadotropin-releasing hormone; MC, menstrual cycle.

Transgender people are a diverse population whose assigned sex at birth are different from their current gender identity. The global prevalence of people who identify as transgender is estimated as 0.3-0.5%, which depends on the definition of transgender used (173). Many transgender people are suffering from health inequities such social marginalization, discrimination, stigma, and violence (174). In the past decades, increasing numbers of people with gender dysphoria have sought medical treatments. According to the clinical practice guidelines from World Professional Association for Transgender Health, those treatments consist of puberty suppression, masculinizing or feminizing hormone treatment, and gender-affirming surgery (175). Application of gonadotropin-releasing hormone analog (GnRHa) or estrogen for puberty suppression in adults and adolescents is recommended by the guidelines. However, data on the efficacy and safety, including the possible metabolic dysfunction and hepatotoxicity, are scarce. A study monitoring triptorelin treatment in gender dysphoric adolescents reports that this agent suppresses puberty in most participated gender dysphoric adolescents. No sustained elevations of liver enzymes or creatinine are observed (176). Another study of 28 transgirl adolescents treated with oral estrogen for more than one year reveals that modest breast development can be found in most participants. The BMI, lean body mass percentage, fat percentage, and liver function do not change during two years of estrogen treatment (177). In a European cohort study of 155 transwomen and 233 transmen, testosterone and estradiol levels are not significantly correlated with amenorrhoea in transmen and breast development in transwomen, respectively. Elevations of liver values are rare (< 4%) and transient in most cases (178). Thus, it seems that GAHT with a safe and effective hormone regimen recommended by the guidelines will not induce liver injury. However, a very recent longitudinal cohort study, which incorporates follow up of over 10 years of 624 transwomen and 438 transmen indicates that, transwomen are likely to experience a moderate elevation of ALT and AST following testosterone initiation, while feminizing GAHT is unlikely to induce such changes. Importantly, alcohol abuse and obesity are strongly associated with liver function abnormalities in transgender populations (179). Thus, more clinical trials and basic studies are needed to delineate the molecular pathways that mediate the sex difference in the liver. Although long-term GAHT under the supervision of clinicians and mental health professionals is not likely to induce evident liver injury, we cannot ignore that many transpeople commonly use sex hormones without any medical supervision and the aware of the potential risks, particularly in the developing world (180). Since irregular and high dosages of sex hormones are common in those transpeople, it is important to test the possible hepatotoxicity and hepatoprotection of those hormones in animal models and, if available, from medical records (**Figure 4**).

## Conclusion

Virtually all kinds of liver diseases are strongly linked with hormonal influences (181, 182). Identifying the clinical manifestation and underlying mechanisms of the “sexual dimorphism” is critical for providing hints for the prevention, management, and treatment of those diseases (76). HRT is used to alleviate vasomotor (e.g. hot flushes and night sweats) and vaginal (e.g. dryness and itching) symptoms of females during menopause because of the reduction in estrogen levels. Previously, the use of HRT is cautioned for patients with basic liver disease since it may provoke or worsen cholestasis (183). Other studies, however, have proved that HRT in patients with chronic liver disease was quite safe and efficacious (184–186). In particular, for patients with chronic liver disease and osteoporosis, transdermal HRT and oral calcium/vitamin D supplementation have been the first-line therapy (187, 188). Results of exogenous sex hormone therapy in liver diseases, both in humans and animals, are controversial. As identified by a Cochrane Review, there is no significant beneficial effect of anabolic-androgenic steroids on clinical outcomes (e.g. liver histology, mortality, and liver-related mortality) of patients with ALD (102). Several small clinical trials tried to examine the efficacy of testosterone therapy in males with cirrhosis, but none of them found beneficial outcomes (189, 190). Since androgen receptor signaling has been shown to suppress metastasis of HCC, combined therapy of Sorafenib and agents that enhance the functional expression of androgen receptor may suppress the HCC progression (106). Similar results are reported in HBV-induced HCC because a small chemical compound that can degrade androgen receptor (ASC-J9) successfully reduce tumor foci and volume in a mice model (95). Estrogen therapy and hormone treatment are generally considered to protect against fatty liver, insulin resistance, and diabetes, although this beneficial effect is not equal in males and females (100). Nuclear receptor proteins (e.g. peroxisome proliferator-activated receptors) are possibly the main targets mediating such protection in the liver (191). Importantly, active estrogen metabolites and derivatives, which have limited affinity for ERs, may play fibrosuppressive roles in the liver (192). Thus, this provides novel therapeutic options for patients with cirrhosis and portal hypertension. An unexplored but promising therapy is the clinical use of prolactin since administration with prolactin or prolactin-releasing peptide evidently improves steatosis in mice obesity models (137), and ablation of prolactin receptor increases hepatic triglyceride accumulation (193). More pre-clinical studies and well-designed RCTs are needed to establish the possible therapeutic effects of prolactin on NAFLD or other chronic liver diseases. Perspectives and side-effects of sex/sex-related hormones or their agonists/antagonists in liver diseases therapy are summarized in **Table 3**.

**Table 3:** Perspectives and side-effects of sex/sex-related hormones or their agonists/antagonists in liver diseases therapy.

Sex/sex-related hormone and their agonists/antagonists	Perspectives	Possible side-effects	References
Androgen	Reducing the levels of ALT, AST, body weight, BMI and waist size; Improving lipid profiles; Providing a potential new target for NAFLD treatment; A potential therapeutic target of HCC	Worsening sleep apnea; Causing acne and skin reaction; Stimulating noncancerous prostate growth and existing prostate cancer; Inducing hepatic insulin resistance in female mice	(41, 44, 47, 106, 194, 195)
Androgen receptor agonist	Reducing atherosclerosis, subcutaneous fat mass, and cholesterol levels in ovariectomized female mice	Reducing the estrogen-induced up-regulation of LDLR; Increasing HCC cell growth and apoptotic resistance	(196–198)
Androgen receptor antagonism	Inhibiting HCC; Improving the efficacy of HCC immune-therapy to PD-L1 inhibitor	Causing a temporary hepatotoxic effect	(48, 49, 198)
Estrogen	Reducing hepatic susceptibility to steatosis; Reducing hepatic lipid accumulation and oxidative stress; Increasing the expression of hepatic apolipoprotein; Inhibiting liver inflammation; Amelioration of liver fibrosis and cirrhosis; Decreasing the deposition of type I and III collagen protein, the total hepatic collagen content and MDA; Reversing liver cell destruction, macrophage accumulation and hepatic stellate cell activation; Reducing portal pressure and increasing hepatic blood flow; Inhibiting HCC	Causing hepatotoxicity such as intrahepatic cholestasis in susceptible females during pregnancy; Inducing acute hepatic porphyrias	(53–56, 59–68, 70–73, 199)
Estrogen receptor agonist	Improving lipopolysaccharide-induced acute liver Injury; Ameliorating liver cirrhosis in rats by inhibiting the activation and proliferation of hepatic stellate cells; Ameliorating hepatic steatosis; Ameliorating liver fibrosis and intrahepatic vascular resistance	Unknown	(200–203)
Estrogen receptor antagonism	Unknown	Increasing portal pressure and decreased hepatic blood flow	(67)
Progesterone	Regulating lipophagy to improve steatosis	Inducing metabolic liver injury; Increasing hepatic glucose production *via* the modulation of gluconeogenesis; Inducing DILI in females; Enhancing hepatitis E virus replication; Increasing risk of ICP; Inducing abnormal proliferation and mitosis in liver cells; Contribution of the development and chemoresistance of liver cancer	(80–92, 204)
Progesterone antagonists	Improving steatosis, insulin sensitivity, and adipocyte ballooning in NAFLD mice	Potential liver toxicity (increased levels of corticosterone and transaminase)	(205–207)
GnRH	Alleviating acute hepatic porphyria	Promoting liver fibrosis; Leading to elevated circulating LDL-C levels	(108, 114, 124, 199)
GnRH agonist	Unknown	Elevating serum liver injury-related enzyme; Reducing liver growth in PLD;	(148, 208, 209)
LHRH agonist	Increasing HDL content; Inhibiting HCC	Reactivating hepatitis B virus	(210–212)
FSH	Maintaining the growth of bile duct cells	A negative impact on blood lipid levels; Increasing cholesterol accumulation; Increasing the risk of NAFLD; Leading to the fasting hyperglycemia	(122, 124, 126, 127, 213)
Prolactin	Ameliorating hepatic steatosis; Alleviating injury of the liver and possibly other ethanol-targeted tissues; Restraining HCC growth	Inducing abnormal drug metabolism and causing DILI	(137, 141, 143, 144)

ALT, alanine aminotransferase; AST, aspartate aminotransferase; DILI, drug-induced liver injury; FSH, follicle-stimulating hormone; GnRH, gonadotrophin-releasing hormone; HCC, hepatocellular carcinoma; HDL, high-density lipoprotein; ICP, intrahepatic cholestasis of pregnancy; LHRH, luteinizing-hormone releasing hormone; PD-L1, programmed death-ligand 1; PLD, polycystic liver disease.

Several problems hinder the development of sex and sex-related hormone-based therapy in liver diseases: (1) lack of mechanistic study, particularly the roles of canonical receptor pathway and non-canonical receptor pathway, which provides the detailed information of drug design and adverse effect; (2) lack of study investigating the complicated interplay between sex/sex-related hormones and other hormones, because several source glands do not only secrete sex/sex-related hormones; (3) the involving roles of precipitating factors of liver diseases, such as alcohol abuse, smoking, and obesity, in sex and sex-related hormone-based therapy need further investigation, both in pre-clinical experiments and clinical trials; (4) maximize the alleviative effects and minimize the side effects of synthesized hormones or their derivatives in clinical application are necessary (e.g. ethynyleestradiol has greater side effects than estradiol valerate); (5) well-designed RCT studies are warranted to ensure the efficacy and safety of novel sex and sex-related hormone-based therapy of liver diseases, with special emphasis in the difference caused by biological sex, age, psychiatric status, and menopause.

We cannot ignore the urgent need for clinical study of possible liver injury after GAHT. Although the standards for optimal individual clinical protocols pf GAHT are generally consistent around the world, the implementation of such service is unequal because of health system infrastructure and socio-cultural contexts (214). The large number of transgender populations that meet difficulty in seeking professional medical help for sex hormone recipes must not be overlooked. Developing novel therapeutic agents for over-dose hormone-induced liver injury is critically urgent for those populations.

In conclusion, both clinical and basic studies provide evidence of sexual dimorphism in liver diseases, from acute liver injury to cirrhosis and HCC. Delineating these observations requires a deep understanding of the characters of sex/sex-related hormones in disease initiation and progression. Whether supplementation of a specific hormone can ameliorate liver injury with acceptable side effects require further basic and clinical studies, particularly for transgender people needing GAHT.

## Author Contributions

LX and YY contributed equally to this paper as co-first authors. LX, YY, CX, and JX conceived and drafted the paper. LX, YY, ZC, and XT prepared the tables and figures. BW instructed and revised the manuscript. All authors contributed to the article and approved the submitted version.

## Funding

This research was funded by the National Natural Science Foundation of China under grant 82122009 and 82070606.

## Conflict of Interest

The authors declare that the research was conducted in the absence of any commercial or financial relationships that could be construed as a potential conflict of interest.

## Publisher’s Note

All claims expressed in this article are solely those of the authors and do not necessarily represent those of their affiliated organizations, or those of the publisher, the editors and the reviewers. Any product that may be evaluated in this article, or claim that may be made by its manufacturer, is not guaranteed or endorsed by the publisher.
